# Should ambulatory appendectomy become the standard treatment for acute appendicitis?

**DOI:** 10.1186/s13017-018-0191-4

**Published:** 2018-06-28

**Authors:** Benoit Gignoux, Marie-Cecile Blanchet, Thomas Lanz, Alexandre Vulliez, Mo Saffarini, Hugo Bothorel, Maud Robert, Vincent Frering

**Affiliations:** 1 Department of General, Visceral and Endocrine Surgery, Clinique de la Sauvegarde, Lyon, France; 2 Department of Anesthesiology, Clinique de la Sauvegarde, Lyon, France; 3 Medical Technology, ReSurg SA, ch. de la Vuarpilliere 35, 1260 Nyon, Switzerland; 4 Department of Digestive Surgery, University Hospital Edouard Herriot, Lyon, France

**Keywords:** Laparascopic appendectomy, Complicated appendicitis, Ambulatory surgery, Conventional hospitalization, Appendicolith

## Abstract

**Background:**

Appendectomy is increasingly performed as a ‘short stay’ or ‘ambulatory’ procedure, yet there is no consensus for selection of patients with acute appendicitis for ambulatory surgery (AS). We aimed to compare characteristics and outcomes of complicated and uncomplicated appendectomies performed in ambulatory vs. conventional settings, and to determine factors associated with unexpected re-consultations and re-hospitalizations.

**Methods:**

The authors reviewed a consecutive series of 185 laparoscopic appendectomies. Whenever possible, patients were offered AS, defined as ‘discharge on the same working day.’ Multivariable regressions were performed to determine associations of unexpected re-consultations and re-hospitalizations with surgery type (ambulatory or conventional) and patient characteristics (age, gender, obesity, symptoms, appendicolith, perforations, appendix diameter, serologic results, American Society of Anesthesiologists score, and Saint-Antoine score).

**Results:**

From the initial cohort, 117 patients (63.2%) were eligible for AS, of which 8 had peri- or post-operative contraindications. Therefore, 109 patients (58.9%) were operated by AS, with median length of stay 8.5 h (range, 3.3–20.5). Ambulatory cases had a lower incidence of complications (11.9%) than conventional cases (25.0%) (*p* = 0.029). Uni- and multi-variable regressions revealed that unexpected re-consultations were not significantly associated with any of the pre- or peri-operative variables but that unexpected re-hospitalizations were 4 times more likely for patients with appendicolith (OR, 4.32; *p* = 0.04).

**Conclusions:**

Ambulatory surgery could be considered as a standard procedure for both complicated and uncomplicated acute appendicitis. Appendicolith was found to be an independent risk factor for unexpected re-hospitalization and should therefore trigger closer monitoring.

## Background

Acute appendicitis is the most common indication for emergency surgery worldwide, with a lifetime risk of 8.6% for men and 6.7% for women [1, 2]. Despite the promotion of antibiotic therapy as a non-invasive alternative, laparoscopic appendectomy remains the standard treatment for acute appendicitis [3–5]. It is a common surgery, performed mostly in young healthy patients, with standardized management, operating time under 1 h, low morbidity, and low average length of hospital stay, all of which render it safe to perform in ambulatory settings [6]. Over the past decades, appendectomy has been increasingly performed as ‘short stay’ (< 24 h hospital stay) [7–10] or ‘ambulatory’ (no overnight hospital stay) [6, 11, 12] procedures, with satisfactory outcomes and low complication rates [13–16]. Yet, conventional hospitalization (with overnight stay) remains the routine procedure for acute appendicitis [6, 16].

There is no present consensus on the selection of patients with acute appendicitis for ambulatory surgery (AS). Recently, Lefrançois et al. [6] proposed a new score, which classifies patients into 6 risk levels, to select patients for ambulatory appendectomy without compromising outcomes. In their series, only 20% were discharged on the same day of surgery. The percentage of appendectomy procedures performed in ambulatory settings reported in the literature varies greatly (20–88%) [6, 8, 11–13, 15–18], likely due to inconsistent definitions of AS and to different criteria for enrollment of ambulatory patients.

For over 5 years, the authors have been performing ambulatory surgery for increasingly complex procedures, including colectomy [19], and have implemented a standardized care protocol for appendectomy following the guidelines of AS for common elective procedures [20]. The primary goal of the present study was to evaluate a consecutive series of appendectomies, complicated and uncomplicated, performed at our center and compare characteristics and outcomes of patients operated in ambulatory vs. conventional settings. The secondary goal was to investigate factors associated with unexpected re-consultations and re-hospitalizations, and thereby improve patient selection for ambulatory appendectomy.

## Methods

The authors retrospectively reviewed a consecutive series of 185 patients who underwent emergency laparoscopic appendectomy for acute appendicitis by 5 surgeons, between January 2015 and June 2016. The series comprised 104 men and 81 women aged 34.1 ± 18.2 (median, 28; range 8–87) at the index operation. Acute appendicitis was suspected on the basis of clinical signs (pain, abdominal tenderness, and muscle guarding in the right lower quadrant), laboratory test results (abnormal white blood cell (WBC) count and serum C-reactive protein (CRP) level), and systematically confirmed by ultrasound (53%) and/or Computed Tomography (CT) (47%).

Following the definition of the International Association for Ambulatory Surgery (IAAS), we defined true AS as ‘a procedure where the patient is discharged on the same working day,’ that is, a procedure with no overnight stay. Ambulatory appendectomy was offered to all patients diagnosed with uncomplicated or complicated acute appendicitis, who presented none of the following *pre-operative* contraindications: (i) cases that could not be operated before 5 pm and could not be postponed to the next day (ii) home-hospital journey over 1 h, (iii) patient living alone, and (iv) severe comorbidities which required monitoring. Furthermore, based on the experience of the emergency team, surgeons reverted to conventional appendectomy in the event of one or more of the following *peri*- and *post-operative* contraindications: (v) generalized peritonitis or abscess requiring administration of intravenous antibiotics (vi) anaphylaxis, (vii) enduring pain that could not be treated by oral analgesic, (viii) fever, or (ix) vomiting.

### Surgical technique

A standard laparoscopic technique was used for all patients. A 10-mm optic trocar was first introduced at the umbilicus and used for a 12-mmHg pneumoperitoneum inflation followed by the introduction of two 5-mm suprapubic and left iliac fossa ports. After a thorough exploration of the abdominal cavity, the appendix was excised and extracted in a bag through the optic tract. Abdominal drainage was restrictively placed in cases with severe infections. Ropivacaine (40 mL, 200 mg) was applied at each trocar site by infiltration as well as at the level of the diaphragmatic cupolas. General anesthesia was performed according to the recommendations of the Enhanced Recovery After Surgery Society [21]. Upon induction of general anesthesia, a single intravenous dose of amoxicillin plus clavulanic acid (2 g) was injected. We followed the guidelines of the French Society of Anesthesia and Resuscitation Medicine regarding the administration of antibiotics. In addition, for an improved rehabilitation and for preventing post-operative nausea and vomiting, intravenous injections of droperidol (1.25 mg) and dexamethasone (8 mg) were administered [22]. Multimodal analgesics were anticipated from the start of surgery using an anti-hyperalgesic agent (ketamine 20 mg) [23], step 1 analgesics (Nefopam + paracetamol + NSAID unless contra-indicated) and completing local and peritoneal infiltrations by local long-acting analgesics [24]. In case of complicated acute appendicitis, patients were given antibiotics postoperatively for at least 48 h.

### Patient management

Hospital facilities included two theaters dedicated to planned and emergency digestive surgery with wide and flexible admission times. Patients diagnosed late afternoon with uncomplicated appendicitis returned home and were operated on the next day. Patients eligible for AS signed a consent form, which included complete information about appendicitis, surgical procedure, and their commitment to inform the clinic about any post-operative complications. AS patients operated on weekdays and discharged from the theater before 5 pm were admitted to the *ambulatory* recovery unit and discharged on the same day. AS patients operated on weekends, and those operated on weekdays but discharged from the theater after 5 pm, were admitted to the *conventional* recovery unit and discharged on the same day. All AS patients were monitored until full awakening and retained until they fulfilled the clinical discharge criteria for ambulatory procedures [25]. Each patient was contacted 1 day following discharge to verify their status and was invited for a follow-up consultation at 1 month. Out of 185 patients operated, 33 (17.8%) patients could not be re-contacted and did not inform the clinic of any complications. They were therefore assumed to have no unexpected re-consultation, re-hospitalization, or re-operation at any other clinic.

The following information was collected for each patient: age, gender, length of hospital stay, operating time, complications within 30 days from discharge, in addition to any unexpected re-consultation, unexpected re-hospitalization, and unexpected re-operation (Table 1). Complications were classified according to the modified Clavien system [26]. Data was collected as part of routine post-operative follow-up and all patients provided informed consent for the use of their data for research purposes. The institutional review board approval was therefore not required for this study.

**Table 1:** Descriptives of the categoric and continuous variables for ambulatory and conventional cases

	Ambulatory cases (*n* = 109)			Conventional cases (*n* = 76)			*p* value
	Mean ± SD	Median	Range	Mean ± SD	Median	Range	
Pre-operative variables							
Age (years)	27.8 ± 13.8	23.0	(8.0–82.0)	43.2 ± 19.9	42.0	(15.0–87.0)	< 0.001
Appendix diameter (mm)	10.0 ± 3.2	9.0	(5.8–26.0)	11.7 ± 4.2	11.0	(5.0–30.0)	< 0.001
CRP level	27.8 ± 37.3	12.0	(1.0–233.0)	63.7 ± 101.7	21.0	(1.0–485.0)	0.016
WBC	12.8 ± 4.9	13.0	(4.1–23.0)	14.1 ± 5.2	13.4	(6.0–28.0)	0.136
ASA score	1.2 ± 0.5	1.0	(1.0–3.0)	1.3 ± 0.6	1.0	(1.0–3.0)	0.393
SA score	3.8 ± 1.1	4.0	(1.0–5.0)	3.3 ± 1.2	3.0	(0.0–5.0)	0.009
Male gender	57.8%			53.9%			0.653
BMI > 28	7.3%			7.9%			1.000
Symptoms > 48 h	38.5%			54.1%			0.049
Appendix diameter > 10 mm	27.8%			52.0%			0.001
Appendicolith	26.6%			27.6%			1.000
Perforation signs	10.1%			25.0%			0.008
Per-operative variables							
Operation-time (mn)	30.8 ± 12.0	30.0	(14.0–88.0)	37.7 ± 16.5	34.0	(15.0–102.0)	0.004
Post-operative variables							
Unexpected re-consultation	11.9%			22.4%			0.069
Unexpected re-hospitalization	4.6%			9.2%			0.236
Unexpected re-operation	0.9%			4.0%			0.306

### Statistical analysis

Descriptive statistics were used to summarize the data. Shapiro-Wilk tests were used to assess the normality of distributions. For non-Gaussian quantitative data, differences between groups were evaluated using Wilcoxon rank sum tests (Mann-Whitney *U* test). For non-Gaussian categorical data, differences between groups were evaluated using Fisher’s exact tests. Multivariable logistic regression analyses were performed to determine associations between two outcomes (unexpected re-consultations and re-hospitalization) and 12 independent variables (gender, BMI > 28, symptoms > 48 h, appendicolith, perforation signs, ambulatory care, age, appendix diameter, CRP, WBC, American Society of Anesthesiologists (ASA) score, Saint-Antoine score. *p* values < 0.05 were considered statistically significant. Statistical analyses were performed using R version 3.3.2 (R Foundation for Statistical Computing, Vienna, Austria). *p* values < 0.05 were considered statistically significant.

## Results

From the initial cohort of 185 patients, 117 (63.2%) were eligible for AS, of which 8 had peri-operative or post-operative contraindications (3 severe infections, 1 anaphylaxis, 2 generalized peritonitis, 2 excessive pain), which left a total of 109 (58.9%) patients operated in ambulatory settings. Their median length of stay, starting from hospital admission, was 8.5 h (range, 3.3–20.5). Compared to conventional cases, ambulatory cases were significantly younger (27.8 ± 13 vs 43.2 ± 20; *p* < 0.001), with lower preoperative CRP blood levels (27.8 ± 37 vs 63.7 ± 102; *p* = 0.016), smaller appendix diameters (10.0 ± 3.2 vs 11.7 ± 4.2; *p* < 0.001), fewer appendix perforations (10.1 vs 25%; *p* = 0.008), better Saint-Antoine scores (3.8 ± 1.1 vs 3.3 ± 1.2; *p* = 0.009), and shorter operation times (30.8 ± 12 min vs 37.7 ± 16.5 min; *p* = 0.004) (Table 1). Moreover, there were no statistical differences between ambulatory and conventional cases in terms of rates of unexpected re-consultations (11.9 vs 22.4%, *p* = 0.069), re-hospitalizations (4.6 vs 9.2%, *p* = 0.236), and re-operations (0.9 vs 4.0%, *p* = 0.306).

Of the 185 patients operated, postoperative complications were noted in 32 (17.3%), and included excessive pain or fever in 13 (7.0%), wound infections in 8 (4.3%), abdominal abscess in 5 (2.7%), liver abscess in 1 (0.5%), ileus in 2 (1.1%), phlegmon in 1 (0.5%), hematoma in 1 (0.5%), and acute urine retention in 1 (0.5%). The 109 ambulatory cases had a lower incidence of complications (11.9%) than the 76 conventional cases (25.0%) (*p* = 0.029). According to the modified Clavien system, the postoperative complications for ambulatory cases were of grade I in 7 (6.4%) patients and grade II in 6 (5.5%) patients, while for conventional cases, they were of grade I in 11 (14.5%) patients, grade II in 6 (7.9%) patients, and grade IIIb in 2 (2.6%) patients. Among the 32 patients with complications, 12 were re-hospitalized for the following reasons: pain or fever without abscess (*n* = 6), abdominal abscess (*n* = 3), liver abscess (*n* = 1), ileus (*n* = 1), and phlegmon (*n* = 1).

Uni- and multi-variable regressions for the entire cohort (*n* = 185) revealed that unexpected re-consultations were not significantly associated with any of the pre- or peri-operative variables (Table 2) but that unexpected re-hospitalizations were 4 times more likely for patients with appendicolith (OR, 4.32; *p* = 0.04) (Table 3). It is worth noting that among the 12 patients who were re-hospitalized, 7 (58%) had appendicolith, with a median time to rehospitalization of 3 days (range, 2–8).

**Table 2:** Logistic regression analysis of re-consultation incidence associated with patient characteristics

Variable	Univariable			Multivariable		
	Odds ratio	95% CI	*p* value	Odds ratio	95% CI	*p* value
Categoric						
Male gender	1.02	(0.47–2.29)	0.957	1.04	(0.41–2.73)	0.931
BMI > 28	0.38	(0.02–2.01)	0.356	0.43	(0.02–3.18)	0.474
Symptoms > 48 h	1.09	(0.49–2.40)	0.823	0.85	(0.31–2.25)	0.746
Appendix diameter > 10 mm	1.12	(0.49–2.48)	0.777			
Appendicolith	1.44	(0.60–3.27)	0.397	1.66	(0.63–4.21)	0.290
Perforation signs	2.20	(0.83–5.42)	0.096	1.84	(0.46–6.55)	0.360
Ambulatory surgery	0.47	(0.21–1.03)	0.062	0.57	(0.21–1.55)	0.267
Continuous						
Age (years)	1.00	(0.97–1.02)	0.812	1.00	(0.97–1.03)	0.814
Appendix diameter	0.98	(0.87–1.08)	0.720	0.95	(0.81–1.10)	0.540
CRP level	1.00	(0.99–1.01)	0.882	1.00	(0.99–1.01)	0.705
WBC	1.06	(0.98–1.16)	0.126	1.05	(0.96–1.15)	0.295
ASA score	0.82	(0.33–1.69)	0.617	0.90	(0.27–2.66)	0.860
SA score	0.88	(0.62–1.27)	0.491

**Table 3:** Logistic regression analysis of re-hospitalization incidence associated with patient characteristics

Variable	Univariable			Multivariable		
	Odds ratio	95% CI	*p* value	Odds ratio	95% CI	*p* value
Categoric						
Male gender	1.60	(0.49–6.19)	0.454	0.71	(0.15–3.47)	0.659
BMI > 28	1.12	(0.06–6.48)	0.917	1.44	(0.05–17.10)	0.788
Symptoms > 48 h	1.25	(0.38–4.15)	0.709	1.46	(0.32–6.56)	0.618
Appendix diameter > 10 mm	1.19	(0.34–3.90)	0.770			
Appendicolith	4.23	(1.29–14.96)	0.018	4.32	(1.09–19.08)	0.040
Perforation signs	2.83	(0.71–9.69)	0.109	3.23	(0.52–17.78)	0.184
Ambulatory surgery	0.47	(0.14–1.54)	0.218	0.85	(0.17–4.58)	0.842
Continuous						
Age (years)	1.01	(0.98–1.04)	0.559	1.03	(0.98–1.07)	0.174
Appendix diameter	1.01	(0.85–1.15)	0.873	0.95	(0.73–1.17)	0.698
CRP level	1.00	(1.00–1.01)	0.329	1.00	(0.98–1.01)	0.438
WBC	1.08	(0.96–1.22)	0.200	1.08	(0.93–1.24)	0.307
ASA score	0.38	(0.02–1.52)	0.306	0.21	(0.01–1.36)	0.180
SA score	0.81	(0.50–1.37)	0.422			

## Discussion

Studies on appendectomy refer to various labels, definitions, and criteria for ‘ambulatory’ surgery, making it difficult to compare the proportions of procedures performed in true ambulatory settings. Some defined ‘ambulatory’ surgery as ‘outpatient procedures’ with a length of hospital stay < 24 h (with or without overnight stay). Although it seems logical to calculate length of stay from the time of hospital admission [8, 13, 15, 18], many authors calculate it from the end of surgery [6, 12, 13, 15, 17, 18]. Over the last decade, definitions of AS changed from day-case surgery with possible overnight stay [17] to a procedure with no overnight stay [6, 11, 12, 16]. We followed the current guidelines of the IAAS and defined ambulatory surgery as ‘a procedure where the patient is discharged on the same working day.’

In this series, 59% of all appendectomies were performed as ambulatory procedures, which is higher than the rates reported in studies with a similar definition of AS (Table 4), whose IIT population comprised complicated appendicitis [6, 11, 12]. Sabbagh et al. [12], Lefrançois et al. [6], and Grelpois et al. [11] reported ambulatory surgery in 18, 20, and 32% of cases, respectively. Cash et al. [13] and Frazee et al. [15, 18], who discharged patients within 24 h, and whose cohorts with intention to treat (ITT) comprised *only* uncomplicated acute appendicitis, claimed remarkable outpatient rates ranging from 65 to 88%. Our rate of AS is all the more remarkable that 27% of patients presented appendicolith and 16% had perforation signs. In our series, 18% of appendectomies could not be performed in ambulatory settings because they were diagnosed or operated outside standard working hours. This was also observed by Scott et al. [16] who demonstrated that surgeries completed after 8pm had 70% greater odds of retaining the patient overnight.

**Table 4:** ‘Ambulatory’ series for acute appendicitis reported in the literature

Author	Year	Indications		Contraindications^b^	Treated by AS		Re-consult	Re-hosp
		(ITT)	N1	for AS	N2	(N2/N1%)		
Studies on patients with UAA and CAA								
This study	2017	AA	185	Severe^a^ AA	109	59%	11.9%^d^	4.6%^d^
Aubry et al.	2017	AA	194	CAA, ASA ≥ 3, age < 15	89	46%		2%^c^
Grelpois et al.	2016	AA age < 18 ASA < 3	240	CAA	76	32%	13%^d^	4%^d^
LeFrancois et al.	2014	AA	184	CAA	37	20%	5%^d^	3%^d^
Sabbagh et al.	2012	AA	123	CAA, age < 18	22	18%	3.1%^d^	3.1%^d^
Dubois et al.	2010	AA	161	CAA, diabetes or immune disorder, age < 16 or > 65	72	45%	11.1%^d^	
Studies on patient with UAA only								
Scott et al.	2016	UAA age < 18	12,703	CAA	6710	53%		2.2%^d^
Frazee et al.	2016	UAA	563	CAA, age < 17	484	86%		1.2%^d^
Frazee et al.	2014	UAA	345	CAA, age < 17	305	88%		1%^d^
Cash et al.	2012	UAA	153	CAA, age < 18	99	65%		0%^c^

*Abbreviations*: *AA* acute appendicitis, *UAA* uncomplicated acute appendicitis, *CAA* complicated acute appendicitis [including perforated/gangrenous appendicitis or abscess], *ITT* intention to treat population, *ASA* American Society of Anesthesiologists score, *SA* Saint-Antoine score, *AS* ambulatory surgery, *IAAS* International Association for Ambulatory Surgery, *LOS* length of stay [from admission]

^a^Severe infections, anaphylaxis, generalized peritonitis, and excessive pain

^b^All studies considered the following characteristics as contraindications: history of abdominal surgery, patient’s refusal, home > 1 h away, living alone, severe pre-op comorbidities

^c^Of patients admitted for AS

^d^Of patients treated by AS

While there are guidelines for deciding on surgical or non-surgical treatment of acute appendicitis [27], there is no present consensus on medical indications to prefer conventional over ambulatory procedures for acute appendicitis [15], although ambulatory procedures have been shown to decrease post-operative morbidities [20]. In France, only 1.3% of appendectomy procedures in 2015 were performed in ambulatory settings [20]. Most surgeons see conventional hospitalization as the standard and look for patients eligible for AS. By contrast, we consider all patients eligible for AS by default, unless they (i) have severe comorbidities which require monitoring, (ii) cannot be operated before 5 pm and cannot be postponed to the next day, (iii) do not meet ambulatory discharge criteria [25], and/or (iv) have severe infections or complications.

Lefrançois et al. [6] created a score to select patients for AS based on 5 preoperative criteria (BMI < 28 kg/m^2^, preoperative CRP levels < 30 mg/dL, preoperative WBC counts < 15,000/mm^3^, diameters of the appendix ≤ 10 mm, and no radiological signs of perforation). In our series, we found that patients with greater CRP levels, greater appendix diameters, perforation signs as well as older patients were less likely to have AS, but found no differences regarding BMI or WBC counts. Frazee et al. [15] and Cash et al. [13] also demonstrated that, in patients eligible for same-day discharge, pre-existing comorbidities as well as older age were factors that prompted conventional surgery. Scott et al. [16] underlined that the decision to discharge younger, healthier patients from the recovery room is often based on the subjective appreciation of operating surgeons rather than objective medical criteria. If so, then the practice of routine conventional hospitalization after appendectomy should be questioned.

In our series, complications were reported in 25% of conventional cases (Clavien grades I to IIIb) and in 12% of ambulatory cases (Clavien grades I and II). The higher rate of complications among conventional cases could be biased because they included patients with severe comorbidities. Among recent studies that used the definition of the IAAS, only Lefrançois et al. [6] reported the complication rate of 8% in their cohort of uncomplicated acute appendicitis treated by AS. In our series, the higher complication rate of 12% could be because we performed AS in a much larger proportion of patients, of whom 27% had appendicolith and 10% had perforation signs. Importantly, AS did not increase the rate of unexpected re-consultation, re-hospitalization, or re-operation. Our re-consultation, re-hospitalization, and re-operation rates amounted to 12, 4.5, and 1%, respectively, which is analogous to rates reported in recent studies [6, 11, 12, 16], even if our series included a larger proportion of ambulatory cases. Overall, the low rate of complications and re-hospitalizations of AS should challenge the current standard of routine conventional hospitalization for uncomplicated appendicitis. If AS is increasingly recognized as safe for uncomplicated cases, our findings suggest it could also be considered in some complicated cases without severe comorbidities.

Our uni- and multi-variable regressions revealed that no variable, including AS, were significantly associated with unexpected re-consultation. The only variable significantly associated with unexpected re-hospitalization was appendicolith, also known as fecalith, corpolith, or stercolith. Appendicolith, which is composed of firm feces and some mineral deposits, is a well-established risk factor for perforation [28–30], inflammation [31], and failure of antibiotics treatment [32] or post-operative adverse events [31, 33, 34]. In our series, BMI was not associated with unexpected re-consultations or re-hospitalizations. In our experience, the Saint-Antoine score is a good predictor of complicated appendicitis, but is too restrictive for selecting patients for AS, notably because of the criterion of BMI > 28. Our uni- and multi-variable regressions revealed that patients with appendicolith were 4 times more likely to be re-hospitalized. It is worth noting that Lefrançois et al. [6] did not include appendicolith in their multivariable analysis on which they based their Saint-Antoine score. Furthermore, as highlighted by Di Saverio et al. [35], each variable selected in the calculation of the total score was given an equal weight of 1 point instead of adjusting the propensity of each variable to its odds ratio. Interestingly, our series presented comparable proportions of appendicolith in ambulatory and conventional cases, which enabled detection of its associated risks, independently from AS or conventional hospitalization. Given the association of appendicolith with re-hospitalization, the authors recommend that appendicolith should therefore trigger closer monitoring.

Appendicolith is a known risk-factor for non-operative treatment of appendicitis [20, 36, 37], and its surgical treatment is recommended [38]. Before conducting this study, the authors did not expect that appendicolith was also a risk-factor in surgical treatment of appendicitis; therefore, they did not manage patients with appendicolith specifically. Recent studies have identified that retained appendicolith after appendectomy is associated with abscess formation and infection [39–42] and suggest that risk of spillage of appendicolith may be reduced by ensuring that the distal end of the appendix is intact after division [41] and by performing a double ligature of appendicular base [43]. Our findings suggest that appendicitis with appendicolith should be considered as “complicated,” and therefore be treated with antibiotics post-operatively. Patients with appendicolith must be informed of increased risks of rehospitalization and be monitored regularly after appendectomy whether performed in conventional or ambulatory setting.

The limitations of this study are typical of retrospective investigation. Out of 185 patients operated, we could not re-contact 33 (17.8%) patients, which may have led us to underestimate the number of minor complications. The strengths of the study include a strict definition of AS rendering comparisons with the literature rigorous, a consecutive series, a multi-operators database, and a large inclusion of patients.

## Conclusion

In the present consecutive series of complicated and uncomplicated appendectomies, 59% were performed as true ambulatory procedures, with patients discharged on the same working day. There were no significant differences in rates of unexpected re-consultations, re-hospitalizations, or re-operations between cases performed in ambulatory versus conventional settings. Therefore, ambulatory surgery seems safe and feasible for selected patients with both uncomplicated and complicated acute appendicitis. To better inform patients and manage their expectations, surgeons should be aware that appendicolith is an independent risk factor of unexpected re-hospitalization for appendectomy and should therefore trigger closer monitoring.
